# Novel pili-like surface structures of *Halobacterium salinarum* strain R1 are crucial for surface adhesion

**DOI:** 10.3389/fmicb.2014.00755

**Published:** 2015-01-13

**Authors:** Gerald Losensky, Lucia Vidakovic, Andreas Klingl, Felicitas Pfeifer, Sabrina Fröls

**Affiliations:** ^1^Microbiology and Archaea, Department of Biology, Technische Universität DarmstadtDarmstadt, Germany; ^2^Cell Biology and LOEWE Research Centre for Synthetic Microbiology, Philipps-Universität MarburgMarburg, Germany; ^3^Department of Biology I, Biozentrum, University of MunichPlanegg-Martinsried, Germany

**Keywords:** haloarchaea, deletion mutant, archaellum, *Halobacterium salinarum*, surface adhesion, archaeal type IV pili

## Abstract

It was recently shown that haloarchaeal strains of different genera are able to adhere to surfaces and form surface-attached biofilms. However, the surface structures mediating the adhesion were still unknown. We have identified a novel surface structure with *Halobacterium salinarum* strain R1, crucial for surface adhesion. Electron microscopic studies of surface-attached cells frequently showed pili-like surface structures of two different diameters that were irregularly distributed on the surface. The thinner filaments, 7–8 nm in diameter, represented a so far unobserved novel pili-like structure. Examination of the *Hbt. salinarum* R1 genome identified two putative gene loci (*pil-1* and *pil-2*) encoding type IV pilus biogenesis complexes besides the archaellum encoding *fla* gene locus. Both *pil-1* and *pil-2* were expressed as transcriptional units, and the transcriptional start of *pil-1* was identified. *In silico* analyses revealed that the *pil-1* locus is present with other euryarchaeal genomes whereas the *pil-2* is restricted to haloarchaea. Comparative *real time* qRT-PCR studies indicated that the general transcriptional activity was reduced in adherent vs. planktonic cells. In contrast, the transcription of *pilB1* and *pilB2*, encoding putative type IV pilus assembly ATPases, was induced in comparison to the archaella assembly/motor ATPase (*flaI*) and the ferredoxin gene. Mutant strains were constructed that incurred a *flaI* deletion or *flaI*/*pilB1* gene deletions. The absence of *flaI* caused the loss of the archaella while the additional absence of *pilB1* led to loss of the novel pili-like surface structures. The Δ*flaI*/Δ*pilB1* double mutants showed a 10-fold reduction in surface adhesion compared to the parental strain. Since surface adhesion was not reduced with the non-archaellated Δ*flaI* mutants, the *pil-1* filaments have a distinct function in the adhesion process.

## Introduction

Various filamentous surface structures have been identified in *Archaea* mediating surface attachment or the formation of cell-cell contacts. These are classified into archaeal type IV pili and non-type IV pili structures. The latter group consists of structurally very diverse representatives (Lassak et al., 2012). The SM1 euryarchaeon forms prickled filaments referred to as “hami” with a distal hook-like structure connecting the cells in a three-dimensional arrangement with regular distances (Moissl et al., 2005; Henneberger et al., 2006). Surface attached cells of *Methanocaldococcus thermoautotrophicum* show networks of thin filaments with diameters of 5.5 nm, the Mth60 fimbriae (Thoma et al., 2008).

Type IV pili and type IV pili-like structures including the archaella (also called archaeal flagella, Jarrell and Albers, 2012) are present in several euryarchaeotal and crenarchaeotal species. The type IV pili biogenesis complexes of archaeal pili are related to those of bacteria (Peabody et al., 2003). The structural components of the pili (Pil) and archaella (Fla) include the assembly/motor-ATPase PilB/FlaI and the multispanning transmembrane protein PilC/FlaJ. These proteins represent in conjunction with the pilins or archaellins the core components of archaeal type IV pili systems (Pohlschröder et al., 2011). Archaeal type IV pili mediate versatile functions like motility, adhesion to biotic or abiotic surfaces, cell-cell connections, biofilm formation and architecture, as well as DNA-exchange (Lassak et al., 2012). A common adhesion strategy has not been observed for *Archaea* so far. In the case of *Pyrococcus furiosus*, the multifunctional archaella are sufficient to mediate surface adhesion and the formation of cell-cell connections (Näther et al., 2006). Surface adhesion of *Haloferax volcanii* solely depends on pili and does not require the presence of the archaella (Tripepi et al., 2010). For *Methanococcus maripaludis* and *Sulfolobus solfataricus* pili and archaella are both necessary for the attachment to a variety of abiotic surfaces (Zolghadr et al., 2010; Jarrell et al., 2011).

By a screening approach with various haloarchaeal strains we demonstrated that surface adhesion is found with the genera *Halobacterium, Haloferax, Halorubrum*, and *Halohasta*. Different type strains, their derivatives and natural isolates of *Halobacterium salinarum* are able to adhere to abiotic surfaces, which supports the subsequent formation of biofilms. Initial studies by transmission electron microscopy (TEM) of surface attached *Hbt. salinarum* DSM 3754^T^ cells showed various filamentous structures on the surface (Fröls et al., 2012). However, the structures mediating surface adhesion are still unknown. In the case of *Hbt. salinarum*, the archaella are the only surface structures described to date (Alam and Oesterhelt, 1984). These filaments are 10 nm in diameter, polarly localized and enable the cells to swim by an ATP-driven rotation (Alam and Oesterhelt, 1984; Cohen-Krausz and Trachtenberg, 2002; Streif et al., 2008).

The aim of the present work was to identify the filamentous structures involved in surface adhesion of *Hbt. salinarum* strain R1 cells *in vitro* and *in vivo*. TEM analyses were used to identify and classify filamentous structures present with surface attached cells of *Hbt. salinarum* R1. A novel type of filamentous structures was observed. The genome sequence was analyzed to search for putative type IV pili gene loci encoding filamentous surface structures other than archaella and two putative type IV pilus biogenesis (*pil*) gene loci were identified. The transcripts were determined and the transcriptional activity of the assembly/motor-ATPases *pilB/flaI* genes was examined by comparative *real time* qRT-PCR analyses in planktonic and surface attached cells. Deletion mutants were constructed to investigate the presence of filamentous structures in dependency of *flaI* and *pilB1* as well as elucidating their role with regard to motility and surface adhesion.

## Materials and methods

### Strains and growth conditions

*Hbt. salinarum* strains R1, DSM 3754^T^, PHH1, PHH4, SB3, GN101, and NRC-1 (strain details were listed in Table 1) were grown aerobically at 37°C in complex medium (250 g NaCl, 20 g MgSO_4_ × 7H_2_O, 2 g KCl, 15 g Oxoid peptone, 50 ml 1 M Tris/HCl pH 7.5 per liter). For cultivation of planktonic and adherent cells an overnight culture with an optical density at 600 nm (OD_600_) of 0.3 was used for inoculation. Before growth the OD_600_ was set to 0.002. Planktonic cells were grown in cultures shaking at 180 rpm and harvested during the exponential growth phase at OD_600_ 0.3 for RNA preparation and OD_600_ 0.5 for DNA preparation. Adherent cells were grown in large Petri dishes (150/20 mm, Sarstedt) as static cultures. After 6 days of growth the supernatant was discarded and the dishes were washed three times with 50 mL salt water (complex medium without peptone) to remove non-adhering cells. Adherent cells were scraped off the dishes using a spatula.

**Table 1:** **Strains and plasmids used for the studies**.

**Strain**	**Origin**	**References**
*Hbt. salinarum* R1 (DSM 671, ATCC 2934)	Derived from DSM670	Pfeiffer et al., 2008b
*Hbt. salinarum* DSM 3754^T^ (ATCC 33171)	Type strain	Elazari-Volcani 1957 (Gruber et al., 2004).
*Hbt. salinarum* PHH4	Derived from PHH1	Pfeifer and Blaseio, 1989
*Hbt. salinarum* SB3	Natural isolate	Ebert et al., 1984
*Hbt. salinarum* GN101	Natural isolate	Ebert et al., 1984
*Hbt. salinarum* NRC-1 (ATCC 700922)	Type strain	Pfeiffer et al., 2008b
*Hbt. salinarum* PHH1	Derived from DSM670	Pfeifer et al., 1988
*Hbt. salinarum* R1 Δ*flaI*	In frame deletion of OE2380R (1890 bp)	This study
*Hbt. salinarum* R1 Δ*flaI*/Δ*pilB1*	Derived from *Hbt. salinarum* R1 Δ*flaI*	This study
	In frame deletion of OE2215R (1758 bp)	
**Plasmid**	**Relevant characteristics**	**References**
pMKK100	*E. coli/Hbt. salinarum* shuttle plasmid, mevinoline resistance, *bgaH* reporter gene	Koch and Oesterhelt, 2005
pMKK100-Δ*flaI*	Contains fusion fragment of the up- (496 bp) and downstream (440 bp) regions of OE2380R	This study
pMKK100-Δ*pilB1*	Contains fusion fragment of the up- (492 bp) and downstream (601 bp) regions of OE2215R	This study

### Transmission electron microscopy (TEM)

Planktonic *Hbt. salinarum* R1 cells were grown at 37°C to OD_600_ 0.8, fixed with 1% glutaraldehyde for 30 min at room temperature and applied onto carbon coated copper grids (400 mesh, Plano GmbH) for 30 s. For the investigation of adherent cells, carbon coated gold grids (400 mesh, Plano GmbH) were placed in a freshly inoculated culture and incubated in static culture at 42°C for 10 days. Adherent cells were fixed with 2% paraformaldehyde w/v and 1% glutaraldehyde w/v over night at 4°C. Samples were washed eight times with double distilled water to avoid the formation of salt crystals. After removing excess fluid using filter paper, samples were contrasted with 2% uranyl acetate (pH 6, containing maleic acid) for 60 s and stored in a desiccator containing silica gel. The software ImageJ (National Institutes of Health, http://rsb.info.nih.gov/ij/index.html) was used for measuring the diameter in nm of visible surface appendages.

### *In silico* analyses

The somewhat similar sequences (blastn), position-specific iterated (psi-blast) and protein-protein (blastp) BLAST® alignment search tools were used to analyze gene and protein identities, functions and presence with other genomes (NCBI Resource Coordinators, 2014). Additional analyses were performed using HaloLex (Pfeiffer et al., 2008a), the UCSC Archaeal Genome Browser (Schneider et al., 2006; Chan et al., 2012) and SMART (Schultz et al., 1998; Letunic et al., 2012). The predictions of transmembrane helices in proteins were performed using the software TMHMM Server v. 2.0 (Krogh et al., 2001), archaeal class III (type IV pilin-like) signal peptides by use of FlaFind 1.2 (Szabo et al., 2007; Esquivel et al., 2013) and the secondary structures of single stranded nucleic acids by the software mfold (Zuker, 2003).

### RNA preparation

Total RNA was isolated from planktonic and adherent cells by standard acid guanidinium thiocyanate-phenol-chloroform extraction (Chomczynski and Sacchi, 2006). Genomic DNA was removed by treatment with RNase-free DNaseI (# EN0523, Thermo Fisher Scientific) for 4 h at 37°C. Purified RNA was used to generate complementary DNA (cDNA).

### Reverse transcription polymerase chain reaction (RT-PCR)

For transcript mapping 40 μg purified RNA were reversely transcribed into cDNA using Random Hexamer Primers (# SO142, Thermo Fisher Scientific) and RevertAid Reverse Transcriptase (# EP0441, Thermo Fisher Scientific) in a total volume of 160 μL according to the manufacturer's protocol. To investigate co-transcription of neighboring genes, oligonucleotides were designed to amplify fragments encompassing the intergenic region and overlapping adjacent genes (see Table S1 and **Figure 2**). In case of co-transcription, these primers will lead to PCR products using cDNA as template. RT-PCR analysis of *pil-1* was performed using *Taq*/*Pfu*-polymerase mix 19:1 (# EP0702 and # EP0502, Thermo Fisher Scientific) (initial step 300 s at 95°C, 35 cycles of 60 s at 95 °C, 90 s at 54°C to 64°C, 135 s at 72°C, end step 300 s at 72°C) according to the manufacturer's protocol. For analysis of *pil-2* the more sensitive Q5-polymerase (# M0491L, New England Biolabs) was used (initial step 300 s at 98°C, 35 cycles of 10 s at 98°C, 30 s at 49°C to 60°C, 40 s at 72°C, end step 120 s at 72°C). Control reactions were performed using a similar RNA sample without reverse transcription to exclude a possible genomic DNA contamination. PCR was performed to validate the amplicon size and specificity of the oligonucleotides using *Hbt. salinarum* R1 genomic DNA as template.

### Transcription start site determination (ARF-TSS)

The transcription start site (TSS) was determined using the Adaptor-and radioactivity-free (ARF-TSS) method described by Wang et al. (2012). Purified RNA was used to generate first strand cDNA using the *pilB1* gene specific oligonucleotide TSS-pil-1-P1-RT (see Table S1 for oligonucleotide sequences) complementary to a sequence located 160 bases downstream of the annotated start codon of OE2215R. The cDNA was circularized using T4 RNA ligase (# EL0021, Thermo Fisher Scientific) to fuse the 3′- and the 5′-end of the cDNA. The circularized cDNA served as template for PCR using the two diverging oligonucleotides TSS-pil-1-P2-PCR and TSS-pil-1-P3-PCR, binding between the sites of the gene specific oligonucleotide and the TSS. PCR products were inserted into pCR® 2.1-TOPO® using TOPO TA Cloning® Kit for Sequencing (# 450641, Invitrogen) following the protocol of the manufacturer and the resulting constructs were used for sequence analysis with standard M13 oligonucleotides.

### DNA preparation and southern analysis

For preparation of genomic DNA, 2 mL cell culture (OD_600_ 0.5) were sedimented by centrifugation, the cell pellet resuspended in 100 μL salt water and lysed osmotically by the addition of 900 μL TEN-buffer (100 mM NaCl, 1mM EDTA, 20 mM Tris/HCl pH 8.0). Standard phenol/chloroform extraction was performed followed by DNA precipitation using isopropyl alcohol. For Southern analysis 3 μg of genomic DNA cut with *Aat*II were separated on 0.7% agarose gels and blotted on Roti®Nylon membranes (pore size 0.2 μm, Carl Roth GmbH & Co. KG). Southern blots were hybridized in standard hybridization buffer with digoxigenin-labeled DNA-probes and detected by use of Anti-digoxigenin-alkaline phosphatase Fab fragments (# 11093274910, Roche) in combination with the Phototope®-Star Detection Kit (# N7020S, New England Biolabs) according to the manufacturer's protocols. A digoxigenin DNA labeling Kit (# 11277065910, Roche) was used to produce DNA-probes by standard PCR using genomic DNA of *Hbt. salinarum* R1 as template in combination with the following oligonucleotides: pil-1-probe-fwd and pil-1-probe-rev producing a 1541 bp PCR product; pil-2-probe-fwd and pil-2-probe-rev producing a 686 bp PCR product (see Table S1 for oligonucleotide sequences).

### Quantitative reverse transcription polymerase chain reaction (qRT-PCR)

For qRT-PCR 5 μg RNA supplemented with 1 ng of an external standard RNA in a total volume of 20 μL were used to generate the cDNA. External standard RNA (length 1790 nt) was produced by *in vitro* transcription of the *bgaH* gene, using T7 RNA polymerase (# EP0111, Thermo Fisher Scientific) according to the manufacturer's protocol. qRT-PCR analysis was performed using the StepOne™ Real-Time PCR System (Applied Biosystems) and the SensiFast™ SYBR Hi-ROX Kit (# BIO-92005, Bioline) according to the manufacturer's protocol. Using the StepOne™ software v2.0 the ΔΔC_T_-method was applied to calculate relative expression changes of the target genes in adherent cells compared to their expression in planktonic cells (Schmittgen and Livak, 2008). *C*_T_-values were determined by the StepOne™ software (Applied Biosystems). *C*_T_-values of the housekeeping genes *rpoB1* (OE4741R) and *aef2* (OE4729R) were normalized to the external standard *bgaH* to investigate the general transcriptional activity of the cells. *C*_T_-values of the target genes were normalized to the housekeeping gene *rpoB1* (Bleiholder et al., 2012). Samples were examined in triplicates. Control reactions checking for genomic DNA contamination were done as described before.

### Construction of deletion mutants

The construction of deletion mutants was performed using the pop-in/pop-out strategy (Koch and Oesterhelt, 2005). Approximately 500 bp upstream (US) and downstream (DS) of the gene of interest were amplified from genomic DNA (initial step 300 s at 95°C, 30 cycles of 60 s at 95°C, 60 s at 58°C to 72°C, 30 s to 150 s at 72°C, end step 600 s at 72°C) and fused by PCR (initial step 300 s at 95°C, 60 s at 60°C, 60 s at 72°C, 10 cycles of 60 s at 95°C, 60 s at 60°C, 30 s at 72°C, end step 600 s at 72°C). Oligonucleotides used are listed in Table S1. The fused US/DS PCR products were cloned into pMKK100 (Koch and Oesterhelt, 2005). Polyethylenglycol-mediated transformation of *Hbt. salinarum* R1 was carried out (Dyall-Smith, 2008), followed by red-blue screening (6 μg/mL of mevinolin and 40 μg/mL of X-gal). Blue transformants were used to inoculate liquid cultures without mevinolin (to induce the pop-out) for three subsequent cultivations with complex medium and plated on agar media containing 40 μg mL^−1^ X-gal. Red colonies were selected for the absence of the gene of interest and verified at the site of the deletion by PCR using genomic DNA (initial step 300 s at 95°C, 30 cycles of 60 s at 95°C, 60 s at 58°C to 72°C, 30 s to 150 s at 72°C, end step 600 s at 72°C) followed by sequencing with US/DS flanking oligonucleotides (listed in Table S1). The deletion strains and plasmids are listed in Table 1.

### Motility assay

To investigate swimming motility, *Hbt. salinarum* R1 and the two deletion mutants were grown in semi-solid medium containing 0.3% agar (w/v) (Patenge et al., 2001). 10 μL of a liquid culture (OD_600_ 0.3) were placed in the center of the agar surface and incubated over 96 h in the dark at 42°C. The diameter of each motility halo was measured in cm using the software ImageJ (National Institutes of Health, http://rsb.info.nih.gov/ij/index.html). The average motility halo and standard deviation was calculated from 19 replicates per strain.

### Surface adhesion on glass

*Hbt. salinarum* strains were grown in Petri dishes (92/16 mm, Sarstedt) containing 15 mL complex medium inoculated with cells from the exponential growth phase (OD_600_ 0.3–0.5). The starting culture was set to a calculated OD_600_ of 0.002 before the cells were grown at 42°C for 10 days. Coverslips were inserted into the media to allow adherence on glass. Prior the microscopic analyses, overgrown coverslips were washed three times with salt water (complex medium without peptone) to remove all non-adherent cells. Microscopic analyses were performed using a Zeiss Axioskop 2 (camera AxioCam MRm, software AxioVision). The software ImageJ (National Institutes of Health, http://rsb.info.nih.gov/ij/index.html) was used to select (using the color-based thresholding function) and measure (using the analyze particles function) the percentage of surface coverage. The quantifications are based on the surface-attached cells of six independent visual fields of separate coverslips, from at least two independent inoculated cultures. Significances (*p*-values) of the percentage of surface coverage between the parental and mutant strains were calculated by an unpaired, two-tailed *t*-test.

## Results

### Identification of surface structures present with surface attached cells of Hbt. salinarum R1

**Figure 1:**
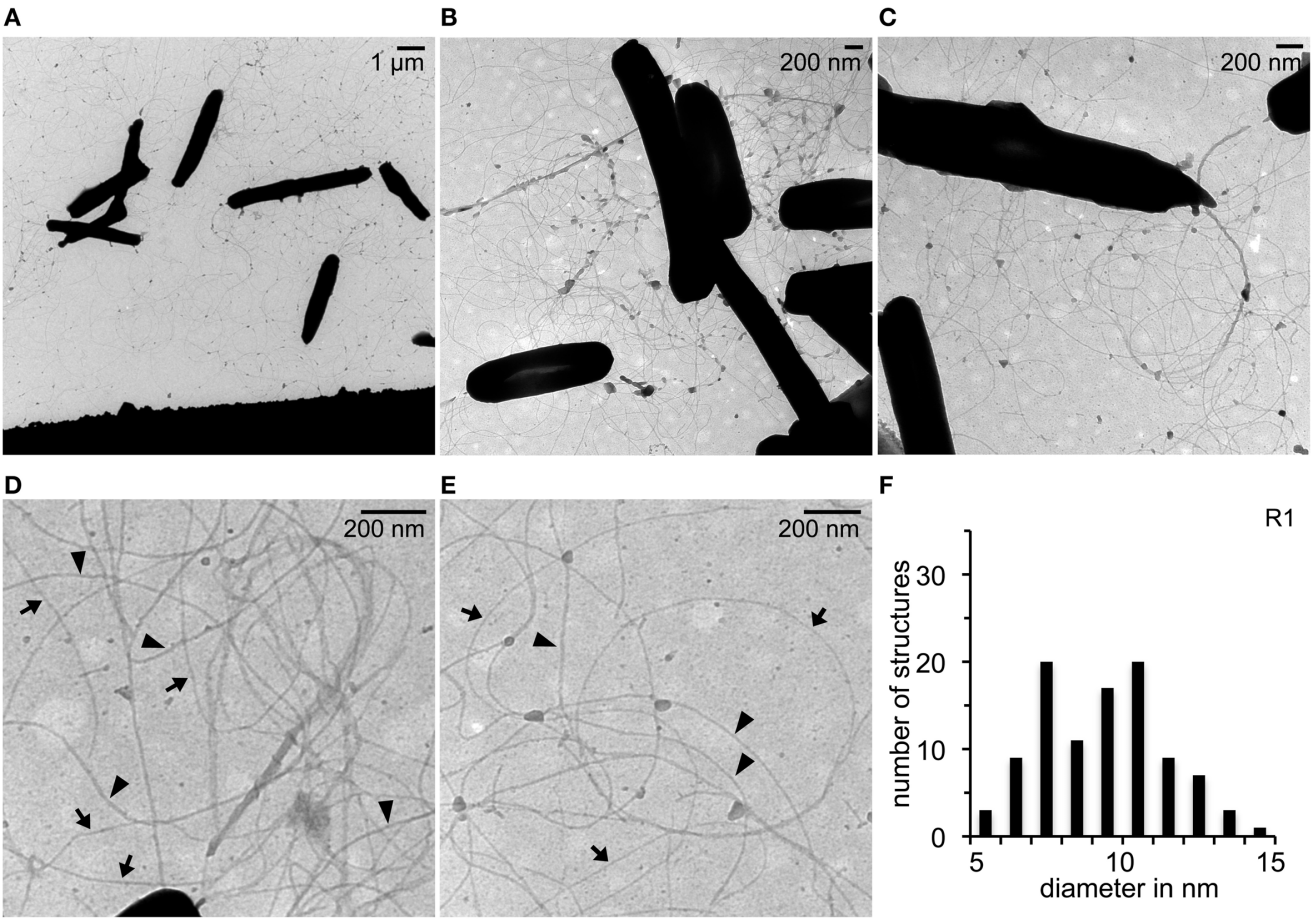
**TEM analyses of *Hbt. salinarum* R1 attached to surfaces. (A)** Surface attached cells grown for 10 d at 42°C on carbon coated gold grids. **(B,C)** Pili-like surface structures observed on the carbon surface. **(D,E)** Determination of different diameters of pili-like structures. Arrow heads, wide diameters; arrows, thinner structures. **(F)** Frequency distribution observed with different diameters determined for 100 surface structures.

TEM was used to investigate the presence of filamentous structures with surface attached cells of *Hbt. salinarum* strain R1 grown on carbon coated gold grids for 10 days (Figure 1A). Numerous long (> 50 nm) and flexible pili-like surface structures were observed between the cells on the carbon surface (Figures 1B,C). The majority of the surface structures were distributed irregularly as single filaments. Many of the filaments originated from the cell poles, but for most of those no origin could be determined. Differences regarding the diameter of single filaments were noticed at higher magnification (Figures 1D,E). The diameters of 100 filaments of at least 10 to 15 photographs were determined using the software ImageJ. The diameters of the filaments were in minimum between 5 and 6 nm and in maximum 14 and 15 nm. The frequency distribution showed two dominant diameters of 7–8 nm and 10–11 nm both with a maximum of 20% (Figure 1F). These data suggested the presence of two different filamentous surface structures in *Hbt. salinarum* R1 with average diameters of 7.6 ± 0.7 nm (calculated on the categories 6 to 9 nm, *n* = 30) and 10.3 ± 0.8 nm (calculated on the categories 9 to 12 nm, *n* = 46). The width of the thicker filaments is consistent with the archaella of *Hbt. salinarum* R1 (Alam and Oesterhelt, 1984; Cohen-Krausz and Trachtenberg, 2002), whereas the thinner filaments might represent a novel pili-like surface structure.

### *In silico* identification and transcription of putative type IV PILI gene loci

The assembly/motor-ATPase FlaI (OE2380R) of the archaellum encoding gene cluster was used as starting point to identify further putative type IV pili biogenesis complex gene loci in the genome sequence of *Hbt. salinarum* R1 (NC_010364.1, Pfeiffer et al., 2008b). By blastp analyses two putative type IV pili assembly ATPases (PilB) homologs of FlaI were identified which share an amino acid identity of 35 and 28% (query coverage 62 and 48%, *e*-value 6e-75 and 5e-21), respectively. These proteins were termed PilB1 (OE2215R) and PilB2 (OE1347R) based on the amino acid identity compared to FlaI. Both proteins contain a conserved VirB11 ATPase domain found with archaeal type IV pili secretion systems (arCOG01817, arCOG01818). The genes *pilB1* and *pilB2* (encoding the putative assembly ATPases) were located adjacent to genes coding for putative multispanning transmembrane archaella/pilus assembly proteins (arCOG01808, arCOG01810) possessing 7 and 9 predicted transmembrane helices. These genes were termed *pilC1* (OE2212R) and *pilC2* (OE1344R), with the latter gene having an in-frame stop codon. The encoded protein PilC1 only has a significant amino acid identity of 23% (query coverage 24%, *e*-value 2e-04) to the archaellar transmembrane protein FlaJ (OE2379R).

**Figure 2:**
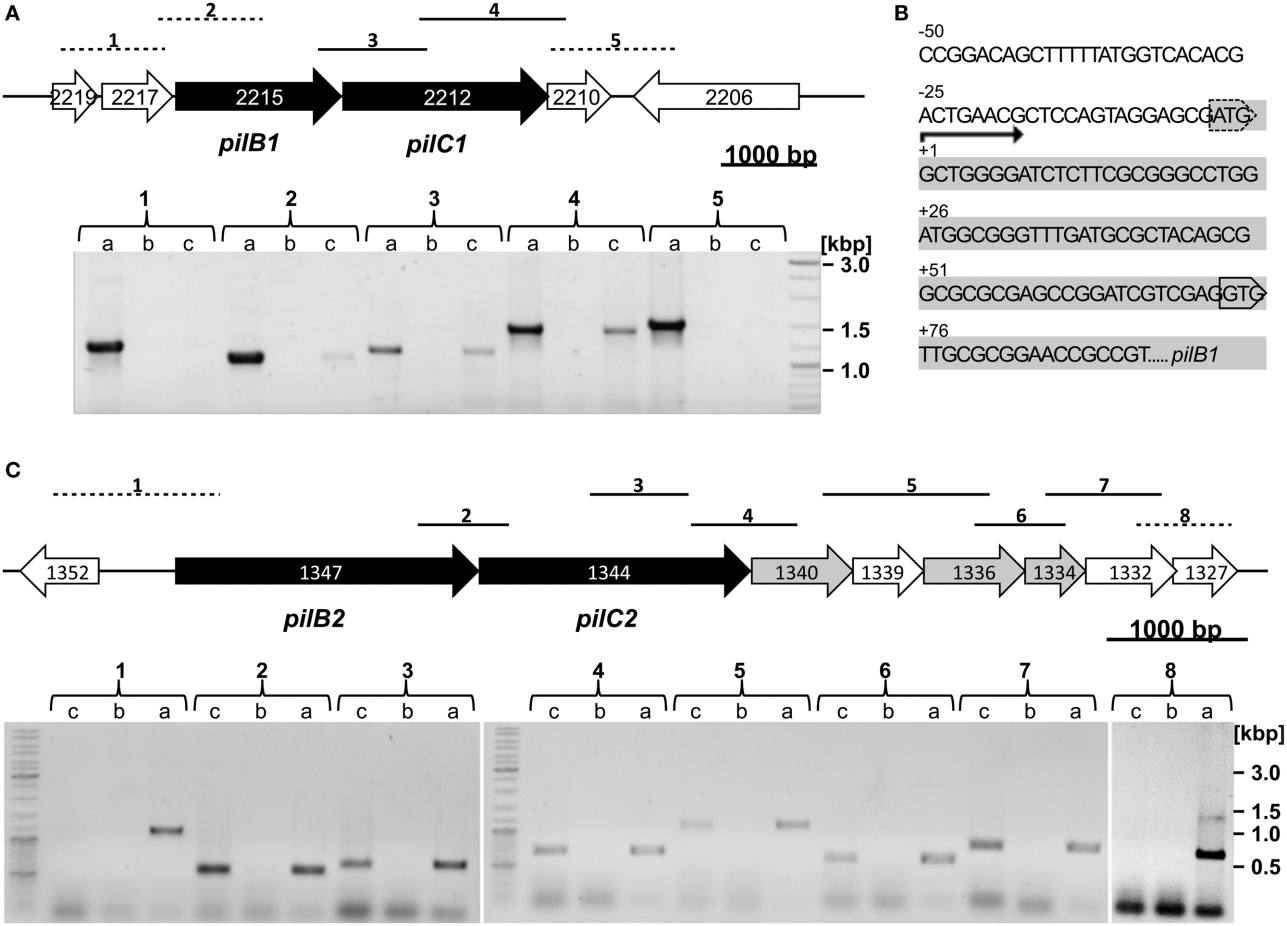
**Genomic regions and transcriptional analyses of the *pil-1* and *pil-2* loci. (A)** *Top: pil-1* locus with genes encoding the putative type IV pili assembly ATPase (*pilB1*) and putative transmembrane protein (*pilC1*) marked in black. *Bottom:* RT-PCR to determine a putative co-transcription using oligonucleotides amplifying fragments across the intergenic regions (brackets numbered 1 to 5 above the gel correspond to fragments 1 to 5 in the gene map; dashed lines, no co-transcription detected; full lines, co-transcription detected). For each pair of adjacent genes the three lanes in the gel represent (a) PCR product using *Hbt. salinarum* R1 genomic DNA as template to validate the amplicon size and oligonucleotides specificity; (b) PCR product with RNA of planktonic *Hbt. salinarum* R1 cells without reverse transcription; (c) RT-PCR product. **(B)** Upstream and 5′ nucleotide sequence of *pilB1* (OE2215R, shown in grey) and the AUG translation start codon predicted for OE2215R is marked by a dashed box. The transcription start site determined by primer extension is labeled +1. The alternative GUG translation start codon is boxed. **(C)** *Top: pil-2* locus with genes encoding the putative type IV pili assembly ATPase (*pilB2*) and putative transmembrane protein (*pilC2*) marked in black. Putative prepilin encoding genes are shown in grey. *Bottom:* RT-PCR experiment investigating co-transcription of the *pil-2* genes similarly to *pil-1* as explained in 2A. Brackets numbered 1 to 8 in the gel correspond to fragments 1 to 8 in the gene map.

Reverse transcriptase polymerase chain reaction analyses (RT-PCR) were performed to determine whether the corresponding genes of the putative type IV pili assembly ATPases *pilB1* (OE2215R), *pilB2* (OE1347R) and putative transmembrane proteins *pilC1* (OE2212R), *pilC2* (OE1344/42R) are transcribed. DNaseI-treated total RNA of *Hbt. salinarum* R1 was used for the generation of the cDNA. No amplification products were observed with RNA, which was not reversely transcribed as template, confirming the absence of DNA contaminations in the sample. Gene specific oligonucleotides were used for the RT-PCR reaction (listed in Table S1) to amplify gene-to-gene overlapping fragments of adjacent genes. The RT-PCR studies confirmed the transcriptional activity of the identified *pilB1, pilC1*, and *pilB2, pilC2* genes (Figures 2A,C). RT-PCR analyses of the gene regions upstream of *pilB1/2* and downstream of *pilC1/2* yielded the transcriptional unit of the putative *pil-1/2* gene loci. The transcript of the *pil*-*1* locus (4.4 kbp) spans the three open reading frames OE2215R, OE2212R, and OE2210R (Figure 2A). Minor or not detectable amplification products were observed for the oligonucleotides combination used to amplify the overlapping gene region of OE2217R and OE2215R. Therefore, the adaptor- and radioactivity-free (ARF) method by Wang et al. (2012) was used to determine the transcriptional start site of the *pil-1* locus (Wang et al., 2012). The sequence analyses of four PCR-products identified the guanine, 4 nt downstream of the predicted AUG as start site the of *pilB1* transcript. A GUG motif as alternative translational start codon is present at position +76 nt, implying the presence of a 5′-untranslated region (5′-UTR) with *pilB1* (Figure 2B). With regard to the *pil-2* locus (6.9 kpb), co-transcription of seven open reading frames, OE1347R through OE1332R, was determined (Figure 2C). These include three genes encoding potential prepilins (OE1340R, OE1336R, OE1334R) containing type IV pilin-like signal peptides as predicted by the software FlaFind 1.2. No potential prepilin encoding genes were found with the *pil*-*1* locus or within the surrounding genomic region of about 100 kbp. A schematic illustration summarizing the results for the *pil*-*1* and *pil*-*2* loci is given in Figure S1.

### Presence of the *pil-1* and *pil-2* gene loci in other haloarchaeal genomes

Southern analyses using gene specific digoxigenin labeled *pilB1/C1* and *pilB2* probes were carried out to investigate the occurrence of the *pil-1* and *pil-2* loci in seven different *Hbt. salinarum* strains. The type strain DSM3754^T^, the three closely related wild type strains R1, NRC-1, PHH1, and the PHH1 derivative PHH4 (strain information is given in Table 1) as well as two natural isolates derived from salt flats in San Francisco (SB3) or Guerrero Negro (GN101) sharing 16S rRNA sequence identities of 98 to 99% to *Hbt. salinarum* R1 but possessing distinct plasmid populations different from the three wild type strains (Ebert et al., 1984). All these species differ in their ability to adhere to surfaces. *Hbt. salinarum* R1 and DSM 3754^T^ show strong, *Hbt. salinarum* PHH4 and SB3 moderate adhesion to a plastic surface, whereas no significant adhesion is observed for *Hbt. salinarum* NRC-1, PHH1, and GN101 (Fröls et al., 2012).

To investigate the presence of the *pil-1* and *pil-2* loci in the genomes of these *Hbt. salinarum* strains, total DNA was isolated and hydrolyzed with the restriction enzyme *Aat*II for Southern analyses (Figure S2A). The *pilB1/C1* probe hybridized with DNA of all strains but strain specific variations were observed (Figure S2B). In five of the seven strains tested (R1, NRC-1, PHH1, PHH4, and SB3) the *pilB1/C1* probe hybridized with two fragments of 4.1 kbp and 3.3 kbp, corresponding to the theoretical sizes calculated from the genome sequence of *Hbt. salinarum* R1 (NC_010364.1). However, only one restriction fragment (3.3 kbp) was detected for *Hbt. salinarum* DSM 3754^T^ and GN101. The *pilB2* probe was expected to label a single fragment with a theoretical size of 4.3 kbp. This fragment was detected in *Hbt. salinarum* R1, DSM 3754^T^, and the natural isolate GN101. *Hbt. salinarum* PHH4 contained a 7 kb fragment, and PHH1 and NRC-1 fragments larger than 10 kbp (Figure S2C). Inspection of the genomic region encoding the *pil-2* locus in NRC-1 (NC_002607) identified a 10 kbp insertion in *pilB2*. The 10 kb insert contains the insertion elements ISH11 (993 bp) and ISH2 (204 bp), and sequence similarities to genes encoding halophage proteins (CopG protein, phage terminase, primase, integrase). The insert inactivates the *pilB2* gene in NRC-1.

Blastn analyses using gene sequences of the transcriptional unit of *pil-1* and *pil-2* were performed to investigate the occurrence of these gene clusters in other archaeal genomes. The analyses indicated that the *pil-1* gene locus (4.4 kbp) is present in a broad range of other representatives of the *Halobacteriaceae* (Table S2). Regarding *Hfx. volcanii* D2 *pil-1* is related to the gene locus encompassing the *pilB3* and *pilC3* genes, required for the PilA pilus biosynthesis (Esquivel and Pohlschröder, 2014). The core unit *pilB1-C1* of the *pil-1* locus is also present in the genomes of methanogenic and hyperthermophilic euryarchaeota but not in the genomes of crenarchaeota or other archaeal phyla. The *pil-2* locus (6.9 kbp) is exclusively found in the genomes of other haloarchaeal strains not with other euryarchaeota (see Table S3). Low identities (query coverage 2% to 7%, *e*-value 8e-05 to 3e-04) were found compared to high GC Gram^+^ actinobacteria, like the biofilm forming *Microbacterium xylanilyticum* (Kim et al., 2005).

### Comparative qRT-PCR of planktonic and surface attached cells

**Figure 3:**
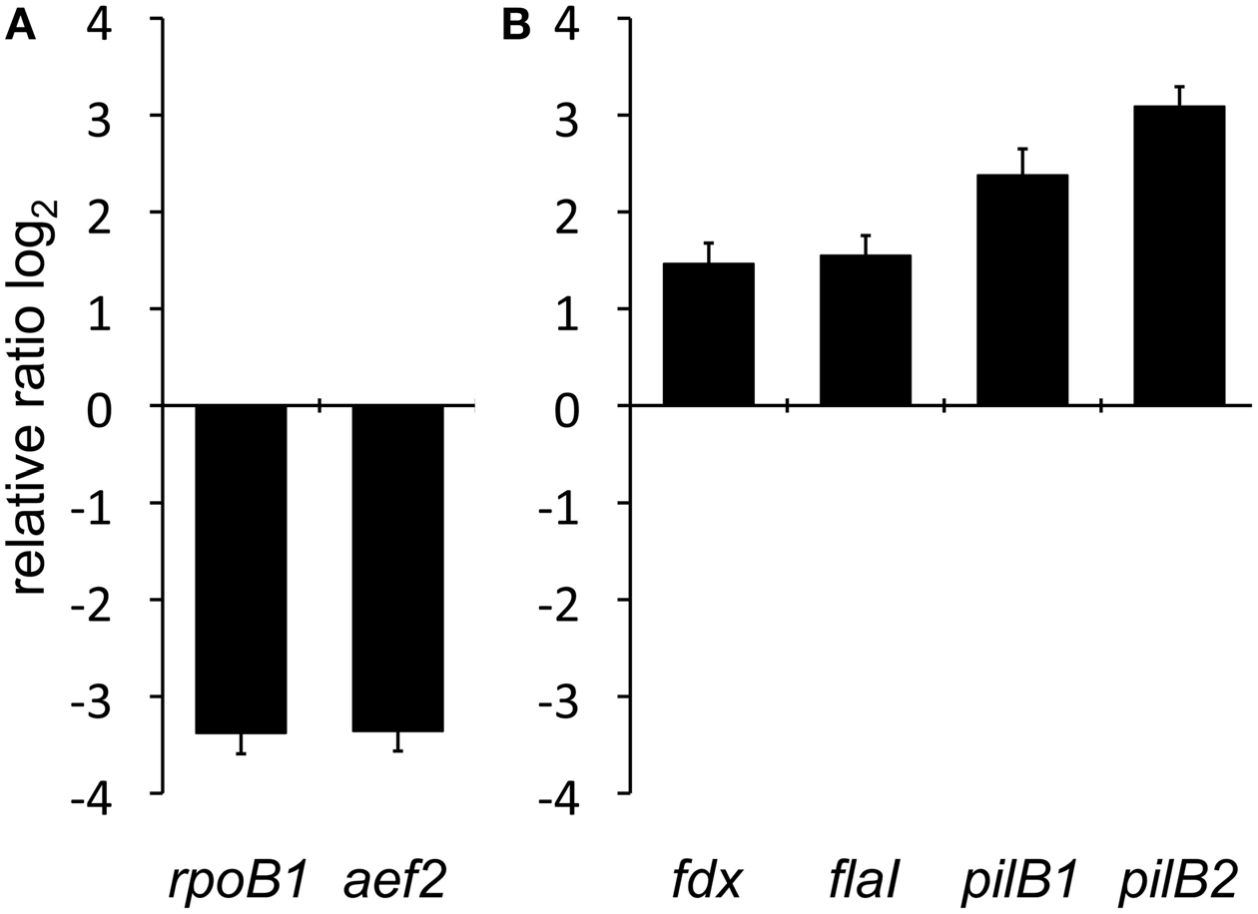
**Comparative qRT-PCR analyses of planktonic and surface attached cells. (A)** Investigation of two representative housekeeping genes encoding RNA polymerase subunit B' (*rpoB1*) and the translation elongation factor 2 (*aef2*). Relative expression was normalized to external standard *bgaH* RNA. **(B)** Relative transcriptional quantification of the assembly ATPase encoding genes of the archaellum (*flaI*) and the type IV pilus biogenesis complexes *pil-1* (*pilB1*) and *pil-2* (*pilB2*) as well as the constitutively expressed ferredoxin gene (*fdx)*. The bars represent the fold change of gene expression shown in base 2 logarithmic scale in adherent cells compared to the planktonic state, which is defined by the baseline.

The expression of the assembly/motor-ATPase encoding genes (*flaI, pilB1*, and *pilB2*) was investigated by quantitative reverse transcription polymerase chain reaction (qRT-PCR) in surface attached cells as well as planktonic cells of *Hbt. salinarum* R1. Total RNA was isolated from planktonic cells during the exponential growth phase (OD_600_ 0.3) and from surface attached cells (grown for 6 days). As control for the cDNA synthesis efficiency *bgaH* RNA (β*-D-galactosidase*, HVO_A0326, *Hfx. volcanii* DS2), was added prior to the cDNA generation and used as an external standard. The oligonucleotides used for qRT-PCR are listed in Table S1. To investigate the general transcriptional activity in surface attached cells two “housekeeping” genes, *rpoB1* (DNA-directed RNA polymerase subunit B', OE4741R) and *aef2* (translation elongation factor, OE4729R), were analyzed by qRT-PCR and normalized to the external control *bgaH* (Figure 3A). For *rpoB1* and *aef2* a 10-fold reduction of the expression was observed in adherent cells compared to planktonic cells. Thus, the overall transcriptional activity is reduced in surface-attached cells of *Hbt. salinarum* R1 after 6 days of incubation. The relative expression of the *pilB1* (OE2215R), *pilB2* (OE1347R), and *flaI* (OE2380R) genes was determined by qRT-PCR to investigate the transcriptional activity of the corresponding gene loci in planktonic vs. surface attached cells, the *rpoB1* was used as internal standard. An induced transcriptional activity was observed for *pilB1, pilB2*, and *flaI* in surface attached cells compared to planktonic cells (Figure 3B). The relative expressions of *pilB1* and *pilB2* were 5.2-fold respectively 8.5-fold enhanced in adherent cells compared to planktonic cells. In contrast, *flaI* showed a 2.9-fold induced transcription in adherent cells compared to planktonic cells which was similar to the gene *fdx* (ferredoxin, OE4217R) with a 2.8-fold induced transcription. Ferredoxin is involved in cellular electron transfer and reported to be constitutively expressed (Twellmeyer et al., 2007). These data indicated a higher transcriptional activity of the *pil-1* and *pil-2* gene loci in surface attached cells. However, the number of cycles for the fluorescence signal to cross the threshold (*C*_T_-value) was for *pilB1* 10 cycles lower (*C*_T_ 21 in surface attached cells) than for *pilB2* (*C*_T_ 31 in surface attached cells), indicating that the transcriptional activity of *pilB1* is higher compared to *pilB2*.

### Construction and phenotypic characterization of Δ*flaI* and Δ*flaI*/Δ*pilB1* mutants

Two deletion mutant strains were constructed from *Hbt. salinarum* R1 to investigate a possible connection between the observed pili-like structures and the induced *pilB1* expression in surface attached cells. The first target for a deletion was *flaI* (OE2380R) to generate cells lacking the archaella, analogous to studies on the archaella encoding *fla*-gene cluster of *Hbt. salinarum* strain S9 showing that the deletion of the assembly ATPase corresponding gene *flaI* is sufficient to obtain non-archaellated cells (Patenge et al., 2001). In the second step the Δ*flaI* mutant strain was used to construct a Δ*flaI*/Δ*pilB1* double mutant (OE2380R/OE2215R) to investigate whether the *pil-1* locus is involved in the biogenesis of the novel pili-like structures. A two step recombination method was used to construct markerless in-frame gene deletions in *Hbt. salinarum* R1 as described in Koch and Oesterhelt (2005). The construction of a Δ*pilB1* single mutant was not successful so far. The deletions of *flaI* and *flaI/pilB1* were identified by PCR using oligonucleotides flanking the genes of interest (Figure S3). The successful deletions were confirmed by sequencing of PCR amplified fragments derived from the deletion mutants, including the regions used for homologous recombination.

**Figure 4:**
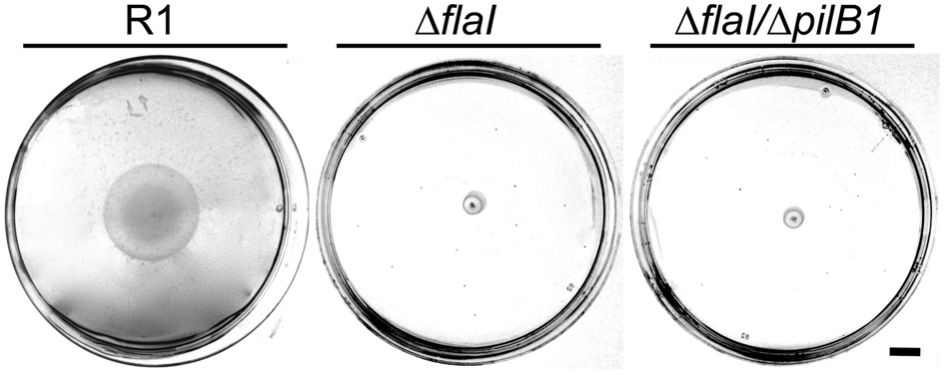
**Motility assay of *Hbt. salinarum* R1, Δ*flaI* and Δ*flaI*/Δ*pilB1* mutant strains**. Swimming motility of *Hbt. salinarum* R1 as well as Δ*flaI* and Δ*flaI*/Δ*pilB1* mutant strains was analyzed in semi solid plates with 0.3% (w/v) agar 3 days after cultivation at 42°C. The scale bar on the right is 1 cm.

**Figure 5:**
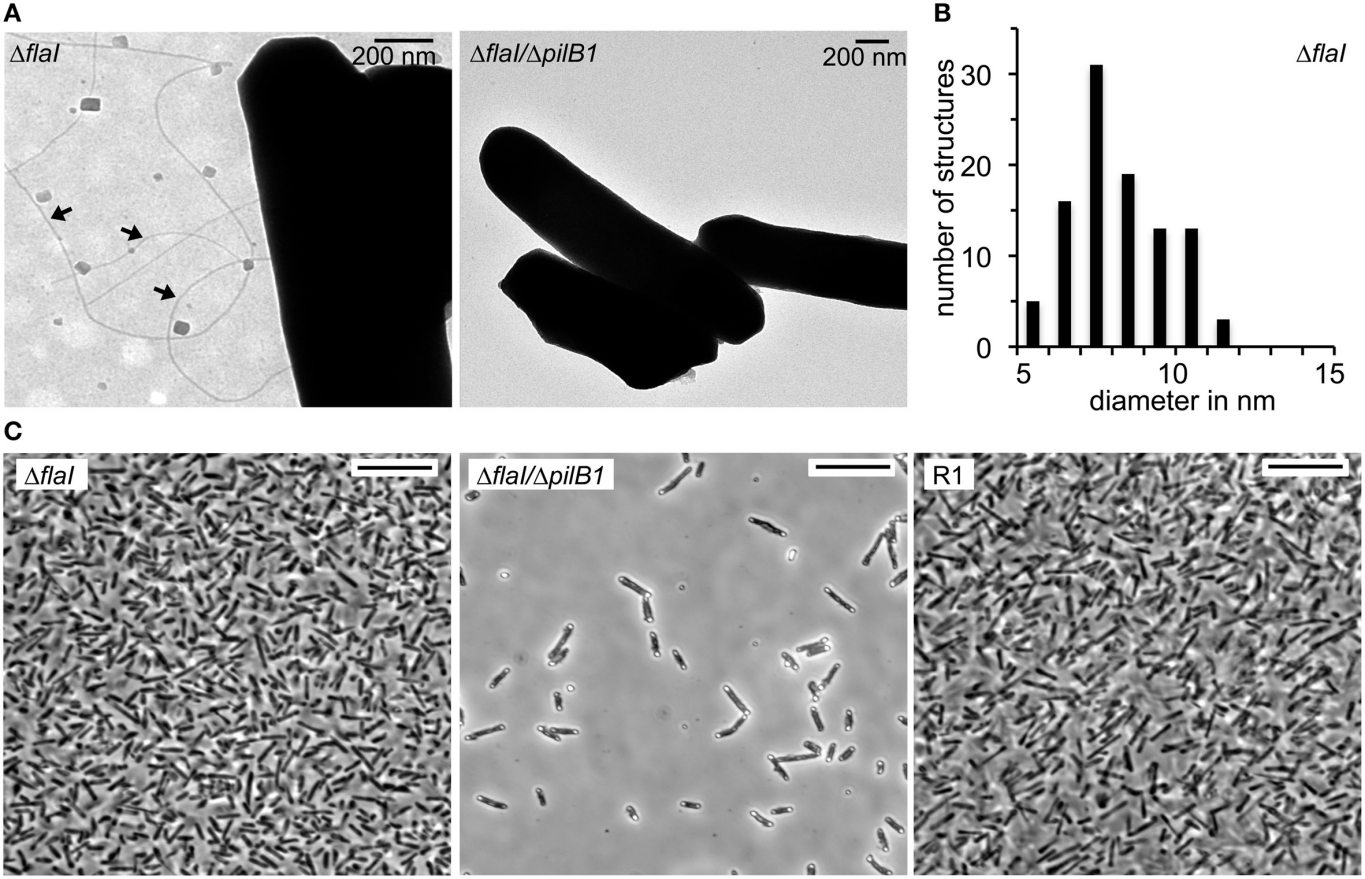
**Phenotypic characterization of the *Hbt. salinarum* R1, Δ*flaI* and Δ*flaI*/Δ*pilB1* mutant strains**. **(A)** Transmission electron micrographs of surface attached cells on carbon coated gold grids after 10 days of cultivation at 42°C. Pili-like surface structures observed with Δ*flaI* are labeled with arrows. **(B)** Frequency distribution of 100 filaments diameters in nm found with the Δ*flaI* mutant. **(C)** Light micrographs of *Hbt. salinarum* R1 and mutant strains attached to a glass surface after 10 days of cultivation at 42°C. Cells not attached to the surface were removed by stringent washing. Scale bars are 10 μm.

The presence of archaella with the parental strain and the deletion mutants was analyzed by a swimming motility assay. Semi solid plates with 0.3% (w/v) agar were inoculated in the center with 10 μL of an exponential culture. The motility halo was measured in cm after 3 days of incubation (Figure 4). Motility was observed with the *Hbt. salinarum* R1 parental strain (motility halo 4.2 ± 1.2 cm, *n* = 19). In contrast, both deletion mutant strains Δ*flaI*/Δ*pilB1* (0.7 ± 0.1 cm, *n* = 19) and Δ*flaI* (0.7 ± 0.2 cm, *n* = 19) did not show any motility, demonstrating that *flaI* is required for swimming. This is in accordance with the data by Patenge et al. (2001). However, with surface attached Δ*flaI* cells pili-like surface structures were observed by electron microscopy (Figure 5A). In comparison to the *Hbt. salinarum* R1 parental strain (see Figure 1) the number of surface structures observed with the cells of the Δ*flaI* mutant were reduced to 10-30%. For planktonic cells of the non-archaellated Δ*flaI* mutant a maximum of 2 to 3 structures were visible per cell (data not shown). The diameters of 100 surface structures found with the Δ*flaI* mutant were determined. The frequency distribution indicated a predominant diameter of 7 to 8 nm found with 31% of the pili-like structures (Figure 5B). The average diameter was calculated to be 7.6 ± 0.8 nm (calculated on the categories 6 to 9 nm, *n* = 66), which is identical to the average diameter determined previously for the novel pili-like structures (Figure 1) of the parental strain. No pili-like structures were observed with any cells of the *ΔflaI/ΔpilB1* mutant, neither with planktonic nor surface attached cells of the entire culture (Figure 5A). These results demonstrated that *pilB1* is required for the assembly of the novel pili-like structures.

To determine a possible role of the novel pili-like structures in surface adhesion, cells of *Hbt. salinarum* R1 parental strain, Δ*flaI* and Δ*flaI*/Δ*pilB1* mutants were grown on cover slips for 10 days. Prior to microscopic analyses the overgrown cover slips were stringently washed to remove all non-surface attached cells. A monolayer of surface attached cells was observed for *Hbt. salinarum* R1 and the Δ*flaI* mutant strains. In contrast, the number of surface attached cells was strongly reduced with the Δ*flaI*/Δ*pilB1* mutant where only a few cells attached to the surface (Figure 5C). To quantify these findings the surface coverage percentage of six photographs was determined for the parental and the mutant strains using ImageJ. The surface coverage of the non-archaellated Δ*flaI* mutants added up to 44% ± 3.6 and was therefore higher [*t*_(12)_ = 3.3, *p* < 0.01] than the value determined for the parental strain *Hbt. salinarum* R1 (36% ± 4.0). The surface coverage observed with the non-archaellated and non-piliated cells of the Δ*flaI*/Δ*pilB1* mutant was 10-fold reduced [4% ± 1.3, *t*_(12)_ = 16.5, *p* < 0.001].

## Discussion

Surface adhesion of the moderate halophile *Hfx. volcanii* DS2 is solely dependent on pili while archaella are not required (Tripepi et al., 2010; Esquivel et al., 2013). For the extremely halophilic archaeon *Hbt. salinarum* structures mediating adhesion have not been described yet. Cells of *Hbt. salinarum* possess polar archaella mediating the swimming motility (Alam and Oesterhelt, 1984). Their possible role in surface adhesion as well as the presence of further surface structures besides archaella were unclear. A first hint for the existence of various filamentous structures came from TEM analyses of surface attached cells of *Hbt. salinarum* DSM 3754^T^ (Fröls et al., 2012).

In this report we determined that *Hbt. salinarum* R1 possesses additional surface structures crucial for surface adhesion. The pili-like structures of 7 to 8 nm are thinner compared to the Aap (archaeal adhesive pili) of *Sulfolobus acidocaldarius* with 10 nm and the type IV pili of *M. maripaludis* with 8.5 nm in diameter (Wang et al., 2008; Henche et al., 2012). Comparison of filamentous surface structures found with surface attached cells of the non-archaellated Δ*flaI* deletion mutant and the parental strain showed that these novel pili-like structures represent approximately one-third of the total cell appendages, illustrating that the archaella are the major surface structures in *Hbt. salinarum* R1. For planktonic cells the total number of these pili-like surface structures is reduced to a maximum of 2 to 3 structures per cell, which explains why the novel structures were not observed before.

Within the genome sequence of *Hbt. salinarum* R1 two putative type IV pili gene loci (*pil-1* and *pil-2*) were present. Transcriptional analyses of the *pil-1* locus identified a 5′-UTR with an alternative GUG translation start codon, which is found in 16% of the genes in *Hbt. salinarum* NRC-1 (Torarinsson et al., 2005). In addition, a putative Shine-Dalgarno element cGAGccGg [consensus sequence for *Hbt. salinarum* GGAGGUGA (Mankin et al., 1985)] was found located 9 nt upstream of the alternative GUG translation start codon, which is in good agreement with the distance for Shine-Dalgarno sequences present in *Hbt. salinarum* PHH1 (Sartorius-Neef and Pfeifer, 2004). Reporter gene studies with the gene encoding the *Hbt. salinarum* PHH1 gas vesicle protein H showed that this SD sequence influences the translation efficiency (Sartorius-Neef and Pfeifer, 2004). In the case of *pil-1* a putative stem-loop structure was predicted for the 5′-UTR, masking the Shine-Dalgarno sequence and the GUG translation start codon, which might effect the *pil-1* translation efficiency, too.

Comparative genome analyses showed that the core unit of the *pil-1* locus is also present in genomes of other euryarcheota and presumably represents a general system for surface adhesion. In contrast, the *pil-2* locus was solely present in haloarchaeal species. It is possible that this region was received from bacteria by lateral gene transfer, since the *pil-2* locus exhibits weak identities with genome sequences of actinobacteria. Comparative analyses of archaeal and bacterial genomes identified 2264 bacterial gene acquisitions by lateral gene transfer in *Archaea* and over 1000 in Haloarchaea, most of them derived from Actinobacteria (Nelson-Sathi et al., 2012, 2014). Representatives of these tolerate the high salt concentrations occurring in saline environments (Hamedi et al., 2013).

Inspection of the particular genomic region in *Hbt. salinarum* NRC-1 identified an additional 10 kbp insert in the *pilB2* gene inactivating the *pil-2* locus. This region represents one of the 12 differences between the chromosomes of *Hbt. salinarum* R1 and NRC-1 (Pfeiffer et al., 2008b) and contains ISH elements and phage-specific sequences. It is flanked by an 8 bp duplication in NRC-1, that is only present as one copy in the *Hbt. salinarum* R1, DL-1, and *Hfx. volcanii* D2, but not with the genomic *pilB2* regions of *Natronomonas pharaonis*, or *Salinarchaeum* sp., suggesting a re-deletion of the region in some of the haloarchaeal strains and the possible reconstitution of *pilB2*.

The results of the quantitative reverse transcription PCR analyses implied a specific transcriptional up-regulation of the *pil-1* and *pil-2* loci in surface attached cells of *Hbt. salinarum* R1. Whether this is regulated directly by the surface contact or indirectly by changing growth conditions (for instance limited oxygen or nutrient supply) is not yet clear. Motility in *Hfx. volcanii* can be altered under certain medium conditions. Archaella-dependent swimming motility is observed with complex or defined medium containing casamino acids but not with defined medium (Tripepi et al., 2010). For *Hbt. salinarum* R1 no significant changes in motility or surface adhesion were observed so far under anaerobic conditions or in response to the reduction of the nutrient source (Völkel and Fröls, unpublished results).

The phenotypic characterization of the Δ*flaI* and Δ*flaI*/Δ*pilB1* deletion mutants indicated that a functional *pil-1* locus is essential for the formation of the novel pili-like filamentous structures. However, additional studies like the constructions of a *pilB1* single deletion strain and its phenotypic characterizations are required, as well as the preparation of the pili structures to analyze their pilin composition. It is questionable which pilins form the surface structure since no putative pilins are encoded in the surrounding genomic region of the *pil-1* locus. Within the genome sequence of *Hbt. salinarum* R1 more than 30 putative pilin encoding genes were predicted (Losensky and Fröls, unpublished results). Which ones of these putative pilins are expressed and assembled into pili structures is not known so far. For *Hfx. volcanii* D2 six pilins (PilA1-6) encoded in different genomic regions were identified. Each of the six pilins alone or in combination is sufficient for the biosynthesis of pili mediating surface adhesion (Esquivel et al., 2013).

The absence of pili-like structures and archaella leads to an impaired surface adhesion phenotype while in the absence of the archaella surface adhesion was increased. We conclude that the pili-like structures and not the archaella are crucial for surface adhesion of *Hbt. salinarum* R1. Nevertheless, we cannot exclude that the archaella are involved in the initial attachment to the surface, as assumed for *S. acidocaldarius* (Henche et al., 2012). In bacteria the flagella are important to mediate the initial reversible attachment to overcome the hydrodynamic boundary layer and repulsive forces. Moreover, a reduced flagellar rotation triggered the transcriptional activation of extracellular polymeric substances by modulation of the second messenger cyclic diguanylate (Petrova and Sauer, 2012).

On the contrary, in the absence of the archaella and pili-like structures low amounts of cells still attached to the surface, indicating that additional surface structures or mechanisms were present. Additional pili-like structures are possibly expressed in minor amounts by the *pil-2* locus, which encompasses three putative pilins. However, due to the internal stop codon within *pilC2* it is questionable whether the *pil-2* locus is functional. Surface adhesion might also be mediated by amyloid adhesins (*i.e*., short fibers formed by an extracellular protein) present with several bacterial biofilm forming species (Larsen et al., 2007). In *Archaea* amyloid proteins are present in the large dense cell clusters and tower like biofilm structures of *Hfx. volcanii* D2 (Chimileski et al., 2014).

Overall the phenotypic characterization of the deletion mutants indicated that the novel pili-like structures are crucial for surface adhesion of *Hbt. salinarum* R1, but the structures themselves still remain to be characterized on the genetic and protein levels.

### Conflict of interest statement

The authors declare that the research was conducted in the absence of any commercial or financial relationships that could be construed as a potential conflict of interest.

## Abbreviations

ARF-TSS: Adaptor-and radioactivity-free identification of transcription start site
Fla: Flagella (archaella) accessory proteins
FlaI: Flagella (archaella) motor/biogenesis protein
Pil: Type IV pilus biogenesis complex
PilB: Type IV pilus biogenesis complex ATPase subunit
PilC: Type IV pilus biogenesis complex membrane subunit
qRT-PCR: Quantitative reverse transcription polymerase chain reaction
RT-PCR: Reverse transcription polymerase chain reaction
TEM: Transmission electron microscopy.
