# Supplementary material for: Novel pili-like surface structures of Halobacterium salinarum strain R1 are crucial for surface adhesion
Source: Front Microbiol. 2015 Jan 13;5:755. doi: 10.3389/fmicb.2014.00755 (PMC4292770; doi:10.3389/fmicb.2014.00755)
Supplement: Supplementary file 1 [file Table1.PDF]

Table S1 Oligonucleotides used for the studies

Name	Oligonucleotide sequence (5'-3')
RT-PCR analyses	
pil-1-RT-1-fwd	GGAGCGACGACTTCACGTAT
pil-1-RT-1-rev	GACGTACAGCGGACGTACAA
pil-1-RT-2-fwd	CACACTCCTCGTCCAGGAAC
pil-1-RT-2-rev	CACCCAGTCGCAGTTCCTC
pil-1-RT-3-fwd	CGATCACCACCAGAATCGT
pil-1-RT-3-rev	TCGCGAAGCGACCTGCTC
pil-1-RT-4-fwd	GGCTCTTGTTGGATTCGATG
pil-1-RT-4-rev	CAGCTTCATGGACGTGGATA
pil-1-RT-5-fwd	GTTCGAAACACCGGTCTCA
pil-1-RT-5-rev	ATGCAGGTACTCACCGTCGT
pil-2-RT-1-fwd	CGCGGTAGACCTCGTTGT
pil-2-RT-1-rev	GATCACCCGCCGGTGTTTG
pil-2-RT-2-fwd	CACCTGCTCCGAGAGATCG
pil-2-RT-2-rev	CACGACACTGCGTACACAC
pil-2-RT-3-fwd	GGTGTTGGTGGCGATCATC
pil-2-RT-3-rev	CCGCAAGCTGGGTATCGAC
pil-2-RT-4-fwd	GGCGACTCTACCACGAGAAG
pil-2-RT-4-rev	GCCGGCGAGATAGGTGA
pil-2-RT-5-fwd	ATCGAAACCACGCACACC
pil-2-RT-5-rev	GATCGTAAGCGACCCTGTG
pil-2-RT-6-fwd	CGACACGCACTCCCAAAC
pil-2-RT-6-rev	TGGTGGTGTCCACACTGAAC
pil-2-RT-7-fwd	CGCGACGATCATCGAGAC
pil-2-RT-7-rev	CTCGCATCGCTTCCCACC
pil-2-RT-8-fwd	GCGTGAGGACAGTGACCA
pil-2-RT-8-rev	ATCGCGGACTCTCCTCTGT
qRT-PCR analyses	
bgaH-qPCR-fwd	TCGTCAAGGGCAAGAACAG
bgaH-qPCR-rev	ACCGAATCCAAGTCGAACAG
rpoB1-qPCR-fwd	GTCCTACGAGGGGTTCAACA
rpoB1-qPCR-rev	CTCGTACGTGCGGAAGAAAT
aef2-qPCR-fwd	GCCGACGAAAGAAGATTGTC
aef2-qPCR-rev	TCAGTGAGGGTGGTTTTTCC
fdx-qPCR-fwd	ACGAGGAAGTCGAGGAGAAGG
fdx-qPCR-rev	CAGGTAGTCGAGGTGCTTGG
flaI-qPCR-fwd	ACGTCCAGGAGCGCATCCT
flaI-qPCR-rev	ACTTGTCGTCGGAGATGCGAA
pilB1-qPCR-fwd	CCGGAAGTACAGCGAGGAG
pilB1-qPCR-rev	GGCTCTTGTTGGATTCGATG
pilB2-qPCR-fwd	CGAAGGTGAACCTCGAACC
pilB2-qPCR-rev	CTTCCGGATGGAGACGTGG
Southern analyses	
pil-1-probe-fwd	CCACACGACGTATTCGACGATGC
pil-1-probe-rev	TCCGTGATCGAGTCC AGGTAGACC
pil-2-probe-fwd	GATGTCCGGGTTGAG GTAGTTG
pil-2-probe-rev	CGTACTACGTGGTGCGTGATCT

ARF-TSS

TSS-pil-1-P1-RT	TCCTCGTAGTCCGCCACC
TSS-pil-1-P2-PCR	GTACGGTGGAACGTCGACG
TSS-pil-1-P3-PCR	CCGCTGGTCGAGTTTGACG

Construction of deletion mutants

flaI-P1	ACGTCTAGAAGCATCTGCTCAACGAGAAC
flaI-P2	GGCTGCCATGTGTATTCCTGTTATGCCAC
flaI-P3	CAGGAATACACATGGCAGCCGAAGCCGAG
flaI-P4	TCGTCTAGACTCTGGTTCCAGGCGTCG
flaI-P5	CTTGGATCCCGACGTCGACACC
flaI-P6	GCGTCTAGAAGCTGCACGATCC
pilB1-P1	TCGCTCTAGATCCGGATCCCGGGCAG
pilB1-P2	CACGCAGTCGCATCTCAGTCACCCGCGTT
pilB1-P3	CTGAGATGCGACTGCGTGGTCGGCGCG
pilB1-P4	TTGTCTAGATCCGTGACCGCGACCGCCA
pilB1-P5	CTTTCCATGGCGCTGAACGCGG
pilB1-P6	GCCAAGCTTCGCAACACCTCGG

Screening of deletion mutants

flaI-Seq-fwd	GCAGCATCGTCCTCGTCGAGG
flaI-Seq-rev	GTTGGCCCTCGTAGGTGGTCG
pilB1-Seq-fwd	TGTGGACCGACACTCATGGTGATGG
pilB1-Seq-rev	GTACTCCTGTTTGAGGAACGCCGG
