# Supplementary material for: Novel pili-like surface structures of Halobacterium salinarum strain R1 are crucial for surface adhesion
Source: Front Microbiol. 2015 Jan 13;5:755. doi: 10.3389/fmicb.2014.00755 (PMC4292770; doi:10.3389/fmicb.2014.00755)
Supplement: Supplementary file 2 [file Table2.PDF]

Table S2 Presence of the *pil-1* locus with archaeal genomes

**Strain**	**Max score**[1]	**OE2215R** *pilB1*	**OE2215R** *pilC1*	**OE2210R**[2] **hyp. protein**	**Comments**
Halobacterium salinarum R1	7999	+	+	+	
Halobacterium sp. NRC-1	7999	+	+	+	
Halobacterium sp. DL1	3056	+	+	+	
Halopiger xanaduensis SH-6	948	+	+	+	
Haloferax volcanii DS2	913	+	+	+	*pilB3/pilC3* in *Hfx. volcanii* DS2
Salinarchaeum sp. Harcht-Bsk1	901	+	+	+[3]	[3] L593_13330 is separated from L593_13350 (*pilC1* homologue)
Halorubrum lacusprofundi ATCC 49239	866	+	+	+	
Halovivax ruber XH-70	861	+	+	+	
Natrinema pellirubrum DSM 15624	847	+	+	-	
Haloarcula hispanica N601	839	+	+	-	DNA-ligase at the third position
Haloarcula hispanica ATCC 33960	839	+	+	-	DNA-ligase at the third position
Halomicrobium mukohataei DSM 12286	838	+	+	-	DNA-ligase at the third position
Haloarcula marismortui ATCC 43049	814	+	+	-	DNA-ligase at the third position
Haloterrigena turkmenica DSM 5511	809	+	+	+	
Haloferax mediterranei ATCC 33500	803	+	+	+	
Haloferax mediterranei ATCC 33500	803	+	+	+	
Natronomonas moolapensis 8.8.11	765	+	+	+	
Halorhabdus utahensis DSM 12940	758	+	+	-	Putative glyoxalase at the third position
Halophilic archaeon DL31	746	+	+	+	
Halogeometricum borinquense DSM 11551	738	+	+	+	
Natronomonas pharaonis DSM 2160	728	+	+	+	
Halorhabdus tiamatea SARL4B	679	+	+	-	Putative encoding glyoxalase gene at the third position
Methanoculleus bourgensis MS2T	354	+	+	-	Hypothetical protein encoding gene at the third position
Methanoregula formicicum SMSP	309	+	+	-	Hypothetical protein encoding gene at the third position
Methanocella arvoryzae MRE50	304	+	+	-	Hypothetical protein encoding gene at the third position
Methanocella conradii HZ254	291	+	+	-	Hypothetical protein encoding gene at the third position
Archaeoglobus fulgidus DSM 4304	145	+	+	-	
Archaeoglobus veneficus SNP6	143	+	+	-	
Archaeoglobus fulgidus DSM 8774	140	+	+	-	
Ferroglobus placidus DSM 10642	98.7	+	-	-	only VirB11-like ATPase present

[1] Blastn analyses were performed using the 4.4 kbp transcriptional unit of the *pil-1* locus identified in *Hbt. salinarum* R1 (NCBI, September 2014).

[2] Probable S-adenosylmethionine-dependent methyltransferase
