# Supplementary material for: Novel pili-like surface structures of Halobacterium salinarum strain R1 are crucial for surface adhesion
Source: Front Microbiol. 2015 Jan 13;5:755. doi: 10.3389/fmicb.2014.00755 (PMC4292770; doi:10.3389/fmicb.2014.00755)
Supplement: Supplementary file 3 [file Table3.PDF]

Table S3 Presence of the *pil-2* locus with archaeal genomes

Strain	Max score[1]	OE1347R *pilB2*	OE1344R *pilC2*	OE1340R[2]	OE1339R	OE1336R[2]	OE1334R[2]	OE1332R	Comments
Halobacterium salinarum R1	12437	+	+	+	+	+	+	+	
Halobacterium sp. NRC-1	9487	+[3]	+	+	+	+	+	+	[3]in NRC-1 *pilB2* homologue is disrupted by a 10 kbp fragment
Halobacterium sp. DL1	2302	+	+	+	+	+	+	+	
Salinarchaeum sp. Harcht-Bsk1	711	+	+	+	+	+	+	+	
Haloferax volcanii DS2	655	+	+	+	+	+	+	+	*pilB1/pilC1* in *Hfx. volcanii* DS2
Halomicrobium mukohataei DSM 12286	650	+	+	+	+	+	+	+[4]	[4] the OE1332R homologue is separated from *pil-2* locus by one ORF
Halorubrum lacusprofundi ATCC 49239	643	+	+	-	-	-	-	-	
Halogeometricum borinquense DSM 11551	621	+	+	+	+	+	+	+	
Natronomonas pharaonis DSM 2160	612	+	+	+	+	+	+	-	
Halophilic archaeon DL31	576	+	+	+	+	+	+	+	
Haloferax mediterranei ATCC 33500	540	+	+	+	+	+	+	+	
Haloarcula marismortui ATCC 43049	484	+	+	-	+	+	+	+	
Natronomonas moolapensis 8.8.11	342	+	+	-	-	-	-	-	
Haloarcula hispanica N601	255	+	+	+	+	+	+	+	

[1] Blastn analyses were performed using the 6.8 kbp transcriptional unit of the *pil-2* the locus identified in *Hbt. salinarum* R1 NCBI, September 2014).
[2] Putative prepilins
