# Supplementary material for: Novel pili-like surface structures of Halobacterium salinarum strain R1 are crucial for surface adhesion
Source: Front Microbiol. 2015 Jan 13;5:755. doi: 10.3389/fmicb.2014.00755 (PMC4292770; doi:10.3389/fmicb.2014.00755)
Supplement: Supplementary file 4 [file Image1.PDF]

Figure S1

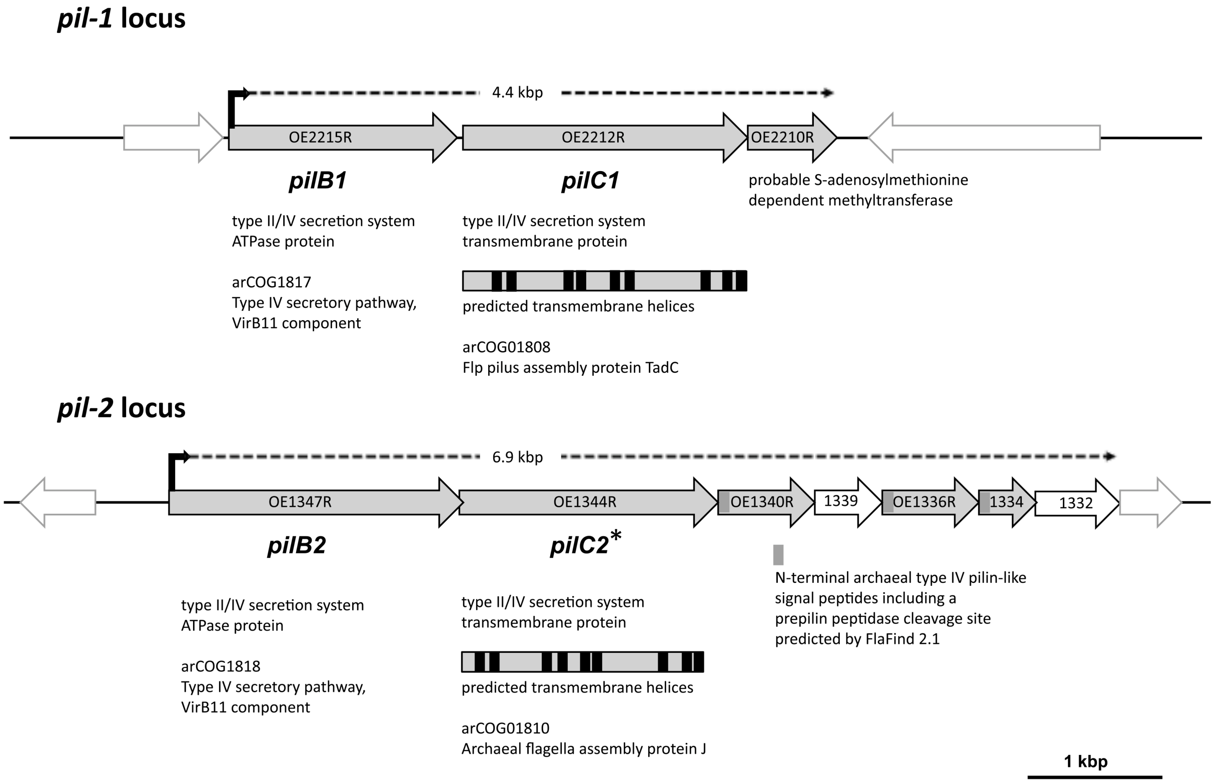

* The putative transmembrane protein encoding ORF OE1344R is interrupted by an internal stop codon (TAG). The same situation is found in the genome sequence of *Hbt. salinarum* NCR-1. For *Hbt. salinarum* DL1 and other close relatives of the genera *Halogranum*, *Halohaptus*, *Haloarcula* and *Halogeometricum* no stop codon is present within the homologous gene region.

Figure S1 Detailed description of the *pil-1* and *pil-2* loci of *Halobacterium salinarum* R1. *Top*: *pil-1* locus of *Hbt. salinarum* R1. Promoter region (arrow) and 4.4 kbp transcript (dashed arrow) comprising three ORFs (OE2215R through OE2210R). *Bottom*: *pil-2* locus of *Hbt. salinarum* R1. Promoter region (arrow) and 6.9 kbp transcript (dashed arrow) comprising seven ORFs (OE1347R through OE1332R). OE1340R, OE1336R and OE1334R might encode prepilins as predicted by their N-terminal signal peptides (dark grey boxes).
