# Supplementary material for: Novel pili-like surface structures of Halobacterium salinarum strain R1 are crucial for surface adhesion
Source: Front Microbiol. 2015 Jan 13;5:755. doi: 10.3389/fmicb.2014.00755 (PMC4292770; doi:10.3389/fmicb.2014.00755)
Supplement: Supplementary file 5 [file Image2.PDF]

Figure S2

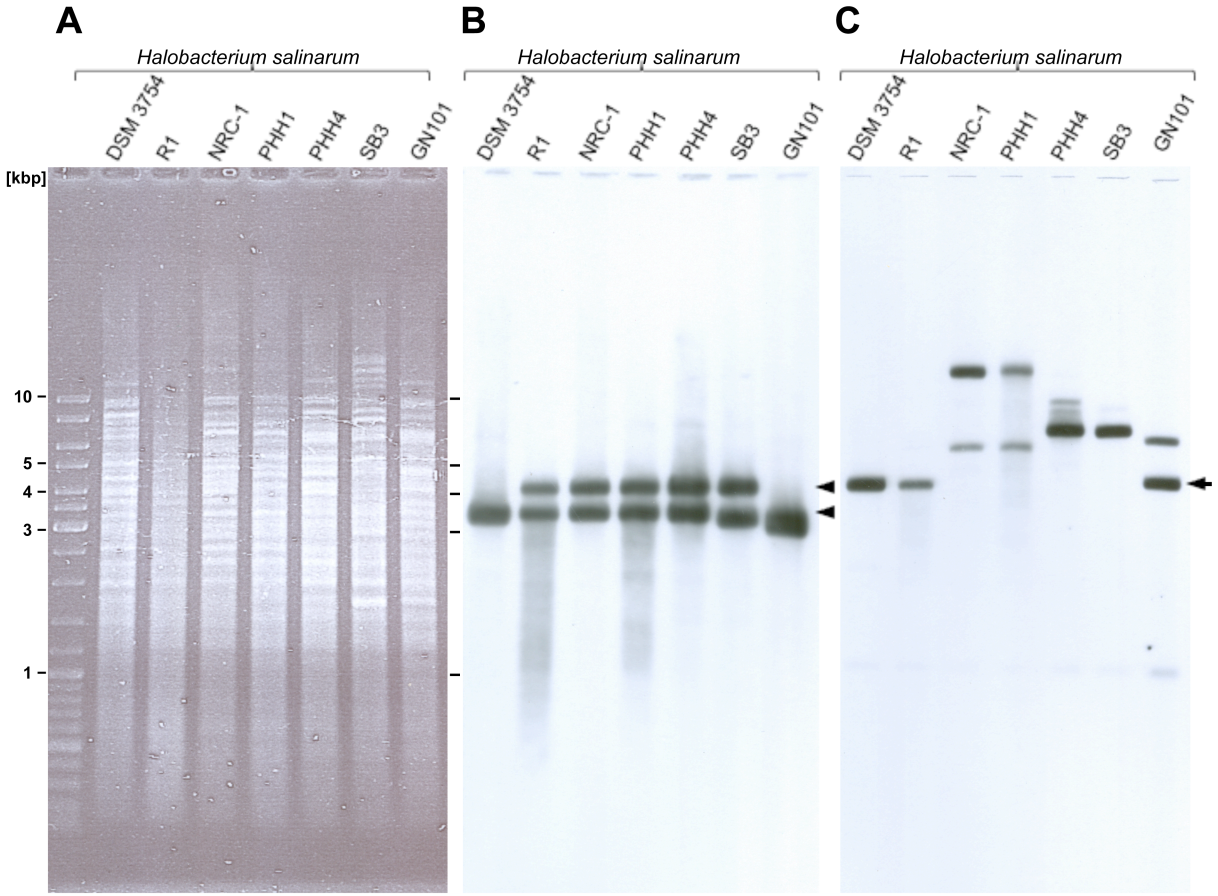

Figure S2 **Southern analysis of different *Halobacterium salinarum* strains investigating the presence of *pil-1* and *pil-2* gene loci.** The strains are described in the results section. **(A)** For each strain 3 µg of total DNA were hydrolyzed with *Aat*II. **(B)** Digoxigenin (DIG) labeled *pilB1/C1* DNA-probes were used for *pil*-1 detection. The expected restriction fragment sizes for *Hbt. salinarum* R1 are 4.1 kbp and 3.3 kbp (arrow heads). **(C)** *pil-2* detection by use of *pilB2* DIG-DNA-probes with an expected fragment size of 4.3 kbp (arrow) for strain *Hbt. salinarum* R1.
