# Supplementary material for: Novel pili-like surface structures of Halobacterium salinarum strain R1 are crucial for surface adhesion
Source: Front Microbiol. 2015 Jan 13;5:755. doi: 10.3389/fmicb.2014.00755 (PMC4292770; doi:10.3389/fmicb.2014.00755)
Supplement: Supplementary file 6 [file Image3.PDF]

Figure S3

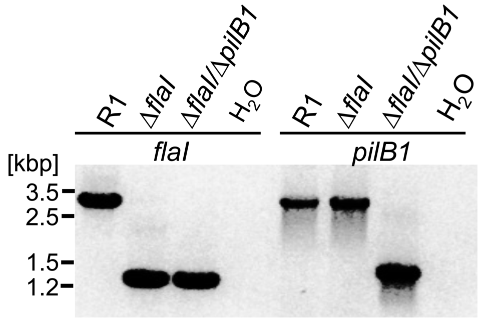

Figure S3 Verification of *Hbt. salinarum* R1, Δ*flaI* and Δ*flaI*/Δ*pilB1* mutant strains.
PCRs using genomic DNA as template were carried out with oligonucleotides flanking the *flaI* and *pilB1* genomic region (listed in Table 1). The absence of *flaI* or *pilB1* genes leads to a reduced fragment size of 1.2 kbp and 1.5 kbp, respectively.
